# Superficial vaginal myofibroblastoma with mushroom-like appearance: A case report with colposcopic findings and literature review

**DOI:** 10.3389/fonc.2022.1024173

**Published:** 2022-10-27

**Authors:** Yibei Wang, Meige Sun, Jiao Wang

**Affiliations:** ^1^ Department of Thoracic Surgery, Shengjing Hospital of China Medical University, Shenyang, Liaoning, China; ^2^ Department of Obstetrics and Gynecology, Shengjing Hospital of China Medical University, Shenyang, Liaoning, China

**Keywords:** superficial myofibroblastoma, vagina, mesenchymal tumor, colposcope, diagnosis

## Abstract

Superficial myofibroblastoma (SMF) of the lower female genital tract is a relatively rare benign mesenchymal tumor. The diagnosis is usually challenging as it shares several similar clinicopathological features with other tumors. Herein, we present a case of a 71-year-old Chinese female patient with postmenopausal vaginal bleeding. Colposcopy imaging revealed a well-circumscribed mass in the vagina with a wide pedicle, resembling a mushroom. The patient underwent surgery, and the tumor was histologically diagnosed as SMF. To the best of our knowledge, this is the first report of colposcopic imaging of a superficial vaginal myofibroblastoma. In this case study, we review the clinicopathological features of SMF of the lower female genital tract reported in the literature to improve the understanding of the disease.

## Introduction

In 2001, Laskin et al. described a distinctive tumor of the cervix and vagina, which they named “superficial cervicovaginal myofibroblastoma (SCVM)” (1). In 2005, Ganesan et al. proposed that the term ‘‘superficial myofibroblastoma (SMF) of the lower female genital tract’’ be used instead of SCVM as some neoplasms have a vulvar location (2, 3). SMFs of the lower female genital tract are an unusual type of benign mesenchymal tumor (4). Only 57 cases have been reported in the literature, among which 43 cases arose in the vagina; the clinical manifestations were not specific, and the lesions were not typical, which makes pre-pathological diagnoses of patients challenging. Here, we present the diagnosis and treatment of a patient with superficial vaginal myofibroblastoma presenting with postmenopausal vaginal bleeding. To further clarify the location, scope, and nature of the lesion, we performed colposcopy on the patient prior to surgery. In addition, we review the literature on the clinicopathological features of this distinctive tumor.

## Case report

**Figure 1:**
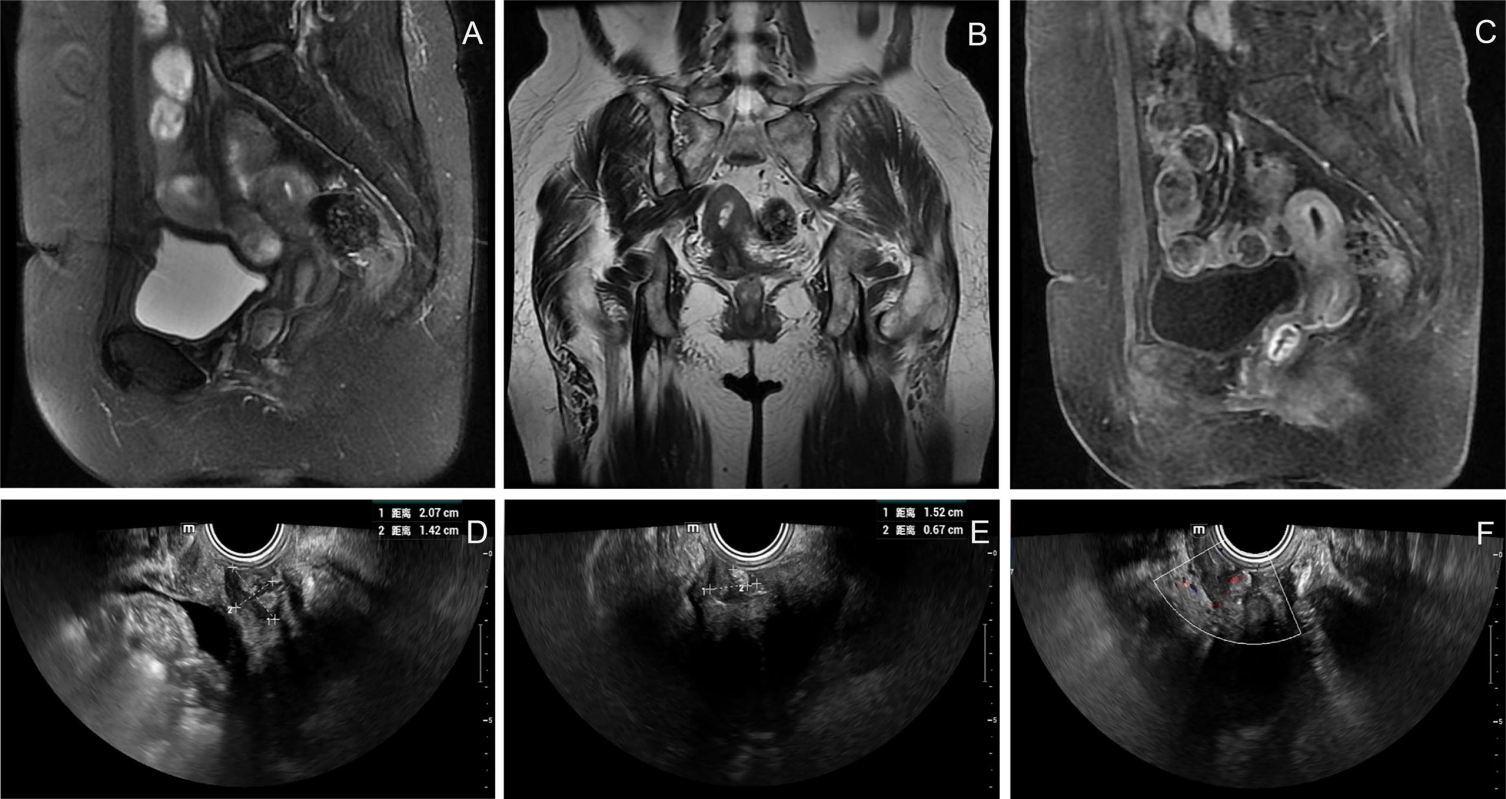
Imaging and ultrasound findings of the patient. **(A–C)** Pelvic enhancement MR: an oval equal T1 mixed T2 signal shadow was observed on the anterior wall of the upper segment of the vagina, approximately 1.7 cm × 1.4 cm × 1.9 cm, with obvious ring reinforcement; **(D–F)** Pelvic ultrasound: a 2.1 cm × 1.5 cm × 1.4 cm mass was observed on the anterior wall of the vagina, with a fuzzy boundary and a hypoechoic inside with multiple strong echoes. CDFI could detect blood flow signals.

The patient was a 71-year-old postmenopausal woman (gravida 2, para 2). On December 17, 2020, the patient visited our hospital for “a small amount of vaginal bleeding for 1 day.” Upon gynecological examination, a 2.0 cm × 2.0 cm mass was found in the upper part of the right wall of the vagina, protruding from the vaginal wall with a smooth surface. On December 22, 2020, a pelvic ultrasound showed endometrial thickening (0.7 cm) and an uneven echo. A 1.2 cm × 0.6 cm × 0.5 cm mass was observed on the anterior wall of the vagina, with a fuzzy boundary, and a hypoechoic inside with multiple strong echoes. Color Doppler flow imaging (CDFI) could detect a few blood flow signals. On December 23, 2020, pelvic enhancement magnetic resonance (MR) was performed, which indicated endometrial thickening and uneven internal signal and enhancement. An oval equal T1 mixed T2 signal shadow was observed on the anterior wall of the upper segment of the vagina, approximately 1.7 cm × 1.4 cm × 1.9 cm, with obvious ring reinforcement (**Figures 1A-C**). We suggested that the patient be hospitalized for surgery, but the patient was not hospitalized in time owing to atrial fibrillation. Therefore, the patient was regularly reviewed using pelvic ultrasound while treating her atrial fibrillation. Ultrasound showed that the vaginal mass gradually increased in size, and there was effusion with a medium echo mass in the uterine cavity. On December 11, 2021, a pelvic ultrasound showed that the thickness of the single-layer endometrium was approximately 0.3 cm, and the echo was uneven. A liquid area with a depth of approximately 0.7 cm was observed in the uterine cavity. The mass on the anterior wall of the vagina grew to be 2.1 cm × 1.5 cm × 1.4 cm in size. CDFI could detect blood flow signals (**Figures 1D-F**).

**Figure 2:**
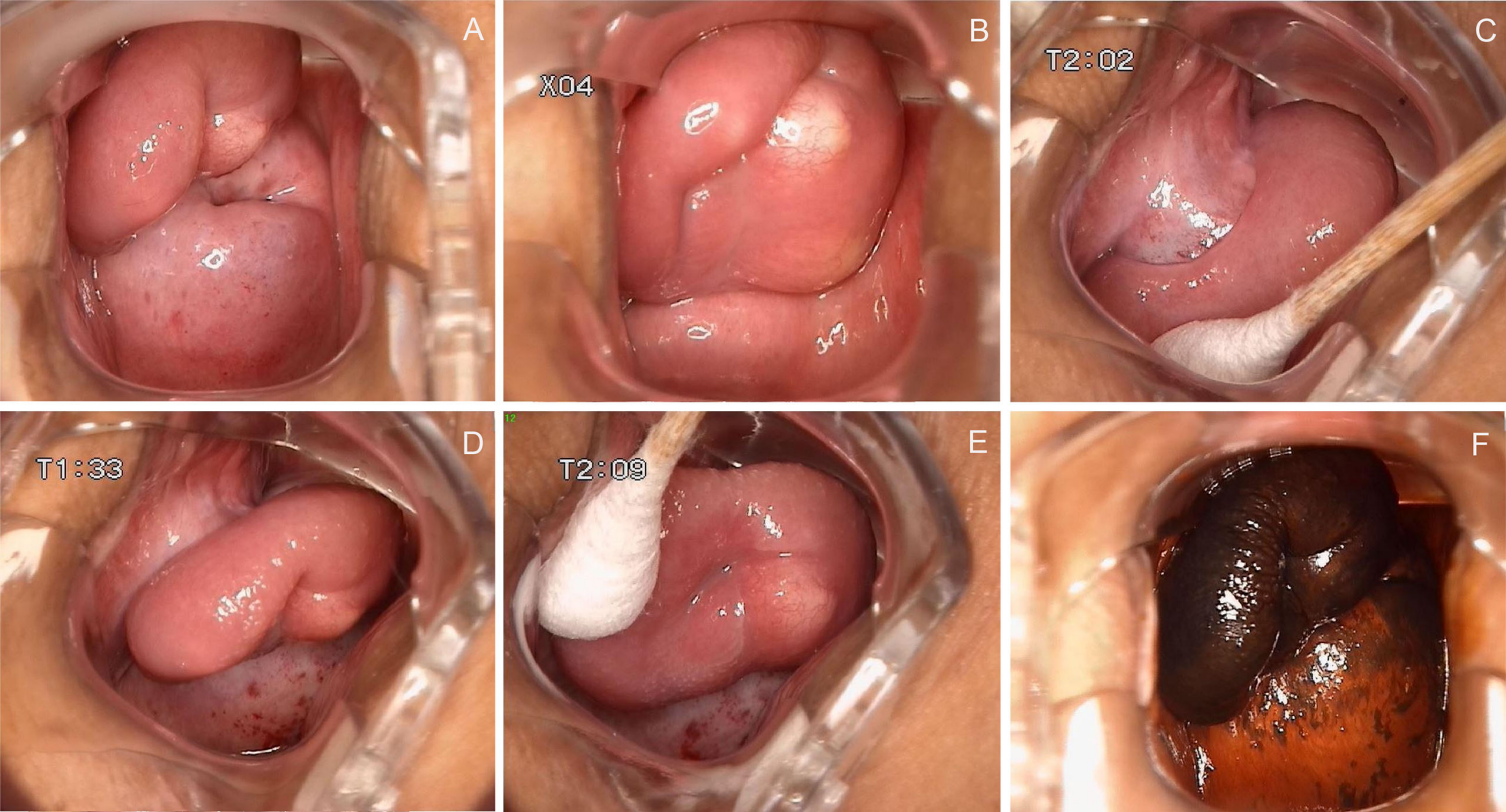
Colposcopy findings of the patient. **(A–C)** A mass was observed on the right anterior wall of the upper part of the vagina, approximately 3.0 cm × 3.0 cm × 1.0 cm in size, pink, tough in texture, smooth on the surface, small vesicles visible inside the mass, a wide pedicle connected to the vaginal wall, and the shape similar to a mushroom; **(D, E)** There was no significant change in the tumor epithelium after staining with 5% acetic acid; **(F)** The tumor epithelium appeared brownish-black after staining with 5% Lugol’s solution.

**Figure 3:**
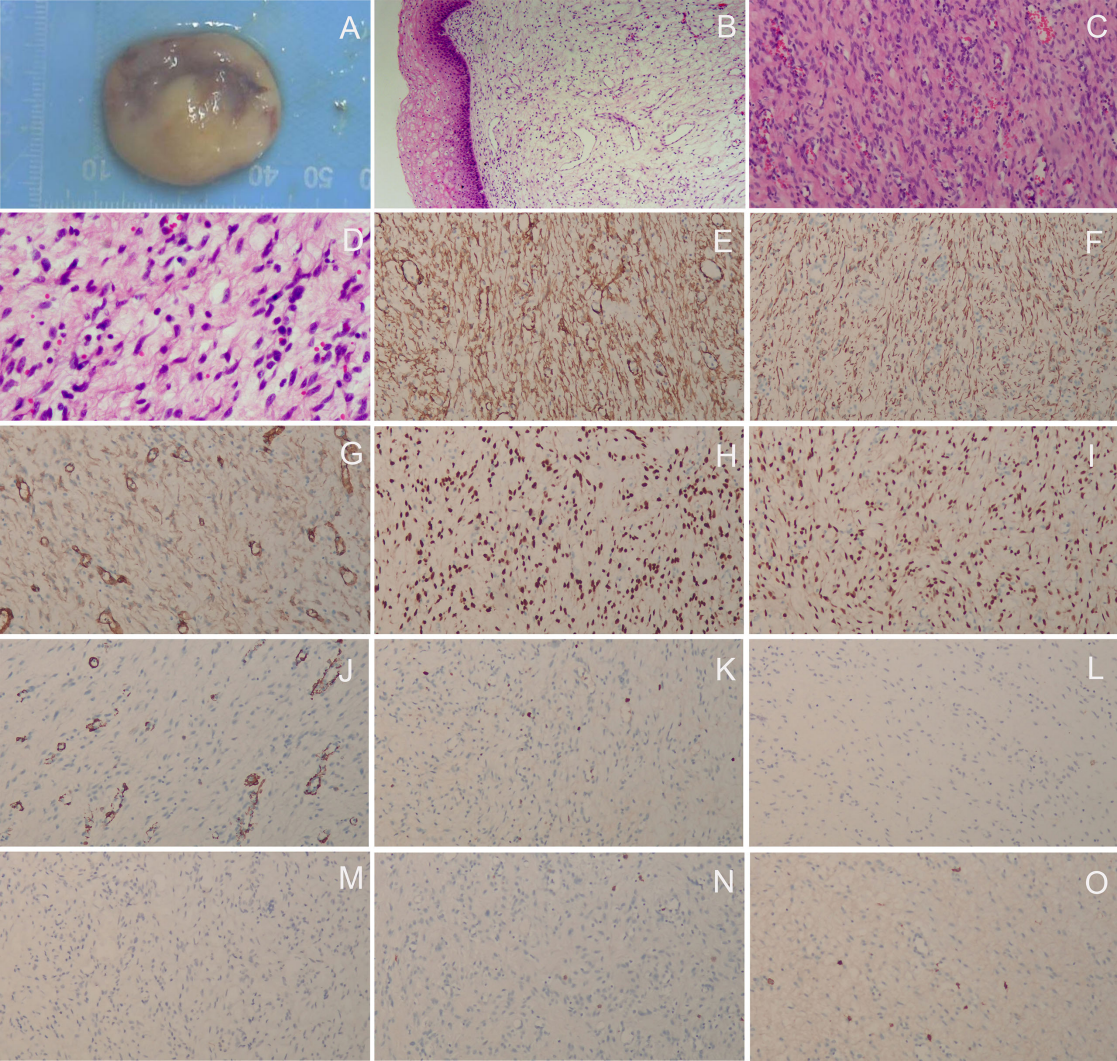
Histopathological and immunohistochemical staining findings. **(A)** The gross pathological examination showed that the size of the tumor was approximately 3.2 cm × 2.8 cm × 0.8 cm, the texture was tough and slightly soft, and the color was pink white; **(B)** The tumor tissue was located under the squamous epithelium (H&E staining, ×40); **(C, D)** The tumor tissue was composed of spindle and stellate-shaped cells and contained abundant thin-walled blood vessels, with some mast cells scattered in the tissue (H&E staining, C, ×100; D, × 200); **(E)** caldesmon (+), **(F)** desmin (+), **(G)** CD34 (+), **(H)** ER (+), **(I)** PR (+), **(J)** αSMA (focally +), **(K)** Ki-67 (approximately 3% +), **(L)** CK (–), **(M)** EMA (–), **(N)** S-100 (–); **(O)** The scattered mast cells were positive for CD117 (**E-O**, ×100).

The patient did not visit our department for hospitalization until June 22, 2022. She was not on any current medications and had no history of tamoxifen or hormonal therapy. On June 23, 2022, the pelvic ultrasound showed that the mass on the anterior wall of the vagina increased to 2.9 cm × 1.7 cm × 1.5 cm in size. Before surgery, we performed a colposcopy to further clarify the location, scope, and nature of the mass. Under the colposcope, a mass was observed on the right anterior wall of the upper part of the vagina, approximately 3.0 cm × 3.0 cm × 1.0 cm in size, pink, tough in texture, smooth on the surface, small vesicles visible inside the mass, a wide pedicle connected to the vaginal wall, and the shape similar to a mushroom (**Figures 2A-C**). There was no significant change in the tumor epithelium after staining with 5% acetic acid (**Figures 2D, E**), and the tumor epithelium appeared brownish-black after staining with 5% Lugol’s solution (an iodine-containing solution) (**Figure 2F**). Therefore, we speculated that this mass was a benign tumor with normal squamous epithelium on its surface. On June 28, 2022, the patient underwent vaginal mass resection and hysteroscopic endometrial polypectomy under general anesthesia. The vaginal mass was completely removed during the surgery, and the frozen section was pathologically determined to be benign. The patient recovered well and was discharged on the third postoperative day. The pathological results of the vaginal wall tumor indicated that it was a superficial vaginal myofibroblastoma. The gross pathological examination showed that the size of the tumor was approximately 3.2 cm × 2.8 cm × 0.8 cm, the texture was tough and slightly soft, and the color was pink white (**Figure 3A**). Microscopic examination revealed that the tumor tissue was located under the squamous epithelium (**Figure 3B**) and was composed of spindle and stellate-shaped cells and contained abundant thin-walled blood vessels, with some mast cells scattered in the tissue (**Figures 3C, D**). Immunohistochemical analysis showed that the tumor was positive for caldesmon, desmin, CD34, estrogen receptor (ER), and progesterone receptor (PR) (**Figures 3E-I**); was focally positive for α-smooth muscle actin (SMA) (**Figure 3J**); and was approximately 3% positive for Ki-67 (**Figure 3K**) and negative for cytokeratin (CK), epithelial membrane antigen (EMA), and S-100 (**Figures 3L-N**). The scattered mast cells were positive for CD117 (**Figure 3O**). No abnormalities were found in the outpatient follow-up 1 month postoperatively.

## Discussion

SMF has been recognized as a mesenchymal neoplasm arising from the superficial portion of the lower genital tract in women (5). The exact etiology and pathogenic mechanisms underlying tumor development remain unclear (5, 6). Liu et al. suggested no association between an SMF tumor and infection with human papilloma virus, Epstein-Barr virus, or human herpesvirus 8 (6). Many gynecologists and pathologists may not be familiar with the disease owing to its rarity. We performed a PubMed-indexed, English-language literature search that yielded 57 reported cases of SMF of the lower female genital tract (**Table 1**). Among them, 75.4% (43/57) occurred in the vagina, 12.3% (7/57) in the cervix, and 12.3% (7/57) in the vulva. The five non-classical myofibroblastoma cases reported by Magro et al. (9) were excluded because it was not clear if the tumors were histologic variants or other types of myofibroblastoma. In the reported cases, 19.5% (8/41) of patients had been taking tamoxifen for whom a history of drug use was available, raising the possibility of a hormone-responsive neoplasm.

**Table 1:** Cases of superficial myofibroblastoma of the lower female genital tract in the English language literature.

Case	Age (yr)	Symptoms	Location	Gross Lesion	Size(cm)	Hormones	Outcome	Follow-up (mon)
1 (1)	40	Asymptomatic	Vagina	Mass	4.5	BCP	NED	120
2 (1)	44	Asymptomatic	Vagina	Polyp	NG	No	NED	240
3 (1)	50	Asymptomatic	Vagina	Polyp	1.3	Tam	NED	48
4 (1)	51	NG	Vagina	Mass	2.0	NG	NG	
5 (1)	54	Asymptomatic	Vagina	Polyp	2.0	No	LTF	
6 (1)	58	Asymptomatic	Vagina	Polyp	1.5	HRT	NED	72
7 (1)	61	Asymptomatic	Vagina	Mass	2.0	HRT+Tam	NED	12
8 (1)	63	Asymptomatic	Vagina	Polyp	1.5	HRT	NED	60
9 (1)	63	NG	Vagina	Mass	1.5	HRT	NED	18
10 (1)	66	NG	Vagina	Polyp	2.0	NG	LTF	
11 (1)	71	Asymptomatic	Vagina	Polyp	1.0	No	NED	12
12 (1)	74	Vaginal bleeding	Vagina	Polyp	6.5	No	NED	60
13 (3)	57	Asymptomatic	Vagina	Nodule	0.2	Tam	NED	14
14 (3)	54	Vaginal bleeding	Vagina	Nodule	0.8 and 0.4	Tam	NED	24 and 12
15 (3)	49	Asymptomatic	Vagina	Nodule	0.4	Tam	NED	24
16 (3)	63	Asymptomatic	Vagina	Polyp	1.4	No	NED	12
17 (3)	80	Asymptomatic	Vagina	Polyp	3.5	No	NED	8
18 (3)	23	Asymptomatic	Vagina	Polyp	4.0	No	NED	8
19 (3)	63	Asymptomatic	Vagina	Polyp	2.7	NG	NED	8
20 (3)	60	Asymptomatic	Vagina	Polyp	1.5	NG	NED	10
21 (3)	62	Asymptomatic	Vagina	Nodule	0.4	NG	NED	11
22 (7)	40	Asymptomatic	Vagina	Polyp or nodule	1.9	No	Recurrence	108
23 (7)	71	Asymptomatic	Vagina	Polyp or nodule	1.9	Tam	NED	6-18
24 (7)	61	Asymptomatic	Vagina	Polyp or nodule	1.6 and 2.2	No	NED	6-18
25 (8)	63	Prolapsed out of vagina	Vagina	Polyp	2.6 and 1.5	Tam	NED	64
26 (8)	47	Asymptomatic	Vagina	Mass	1.5	No	NED	6
27 (8)	54	Prolapsed out of vagina	Vagina	Polyp	3.7	No	NED	4
28 (8)	56	Prolapsed out of vagina	Vagina	Polyp	3.0	No	NED	2
29 (9)	73	NG	Vagina	Polyp or nodule	2.5	No	NG	
30 (9)	69	NG	Vagina	Polyp or nodule	2.0	No	NED	96
31 (9)	44	NG	Vagina	Polyp or nodule	1.5	BCP	NED	11
32 (9)	77	NG	Vagina	Polyp or nodule	0.4	No	NED	6
33 (10)	63	Asymptomatic	Vagina	Polyp	4.0	No	NG	
34 (4)	73	Prolapsed out of vagina	Vagina	Mass	4.7	Tam	NG	
35 (6)	59	Postcoital bleeding	Vagina	Polyp	2.0	No	NED	12
36 (11)	50	Vaginal bleeding	Vagina	Nodule	1.6	No	NED	96
37 (12)	42	Vaginal bleeding	Vagina	Mass	3.2	No	NG	
38^*^	71	Vaginal bleeding	Vagina	Mass	2.9	No	NED	1
39 (5)	40	NG	Vagina	Nodule	5.0	NG	NG	
40 (5)	55	NG	Vagina	Nodule	1.0	NG	NG	
41 (5)	45	NG	Vagina	Nodule	1.1	NG	NG	
42 (5)	60	NG	Vagina	Nodule	1.0	NG	NG	
43 (5)	55	NG	Vagina	Nodule	5.0	NG	NG	
44 (1)	40	Asymptomatic	Cervix	Polyp	4.0	No	NED	36
45 (1)	58	Vaginal discharge and prolapsed out of vagina	Cervix	Polyp	5.0	HRT	NED	48
46 (7)	49	Asymptomatic	Cervix	Fibroid	4.5	No	NED	6-18
47 (13)	27	Prolapsed out of vagina	Cervix	Mass	5.0	No	NG	
48 (14)	45	Menometrorrhagia	Cervix	Mass	3.8	No	NG	
49 (15)	45	Pelvic pain and menometorrhagia	Cervix	Mass	6.5	No	NED	96
50 (5)	74	NG	Cervix	Nodule	3.0	NG	NG	
51 (3)	27	Asymptomatic	Vulva	Mass	2.7	NG	NED	60
52 (3)	38	Asymptomatic	Vulva	Mass	4.5	NG	NG	
53 (9)	NG	NG	Vulva	Mass	1.2	No	NG	
54 (16)	37	Asymptomatic	Vulva	Mass	7.0	NG	NED	12
55 (17)	77	Bulky swelling	Vulva	Mass	12.0	No	NG	
56 (5)	46	NG	Vulva	Nodule	10.0	NG	NG	
57 (5)	26	NG	Vulva	Nodule	6.5	NG	NG	

NG, not given; BCP, birth control pills; Tam, Tamoxifen; HRT, hormone replacement therapy; NED, no evidence of disease; LTF, lost to follow-up. ^*^current case.

The symptoms in patients with SMF were non-specific. The main clinical manifestations were asymptomatic polypoid or nodular masses of varying sizes (0.2–12 cm). Occasionally, the mass may prolapse out of the vagina or manifest as abnormal vaginal bleeding. Only five of the 57 patients had two lesions, and the rest had single lesions. The patients ranged in age from 23–80 years, with a mean age of 54.7 and a median age of 55.5 years. Only six patients were younger than 40 years of age, and only one of them was pregnant. In our case, a vaginal mass was found during gynecological examination due to postmenopausal vaginal bleeding, which may have been caused by endometrial polyps.

The diagnosis of SMF of the lower female genital tract is usually challenging because it is rare and shares many clinicopathological features with other mesenchymal tumors, such as fibroepithelial stromal polyps, angiomyofibroblastoma, mammary-type myofibroblastoma, cellular angiofibroma, and aggressive angiomyxoma (12, 18). The diagnosis should be combined with gynecological examination, ultrasound, and MRI, and the final diagnosis still depends on histopathology. Histological examination shows a well-circumscribed, yet unencapsulated lesion, covered by unremarkable or hyperplastic squamous epithelium (19). Tumors usually present with spindle and stellate-shaped cells within a collagenous stroma, showing an expansive growth pattern with a grenz zone of uninvolved tissue (12). Multiple patterns, including lace-like, sieve-like, and fascicular, are characteristic features, as are myxoid or edematous foci, and few mitotic figures (19). Cells are positive for vimentin and usually for desmin. CD34 and αSMA are positive in some cases, and most neoplasms are positive for ER and PR. Tumors are negative for S100, EMA, and CK (19). The case described in this report is consistent with the above histopathological features, and the histopathological features of other mesenchymal lesions have been described in detail previously (12, 18, 19).

As described in the literature, SMF often presents as a well-circumscribed polypoid or nodular mass. Our case also presented as a nodular mass, and we used colposcopy to magnify the lesion to visualize a more intuitive manifestation of this mass. Simultaneously, we observed a wide pedicle that connected the mass to the vaginal wall, similar to that reported by Tomita et al. (12) and Adams et al. (13). Owing to the small number of cases, it is unclear whether this phenomenon is unique to SMF. To the best of our knowledge, this is the first report of colposcopy as an aid in the diagnosis and evaluation of SMF. Colposcopy is a procedure in which a lighted, magnifying instrument called a colposcope is used to examine the cervix, vagina, and vulva (20). Colposcopy is important in the diagnosis of cervical lesions. It also plays a significant role in the diagnosis of vaginal lesions, because the shape, location, and scope of the lesions can be clearly observed, and the reaction of the lesion epithelium to 5% acetic acid and Lugo’s solution can be applied in this small space. In the present case, no abnormal changes were observed after the use of acetic acid or Lugol’s solution. Dysplastic cells dehydrate and turn densely white with the application of acetic acid (20). Lugol’s solution may also be used to highlight the dysplastic area, where the dysplastic cells remain yellow owing to a lack of absorption of the solution (20). Although definitive diagnosis should be based on histopathological assessment, colposcopic features such as well-defined morphology and coverage with normal squamous epithelium may contribute to differentiating from more aggressive entities such as malignant tumors.

Surgical resection is the main clinical treatment for SMF; among 37 patients with available follow-up information (1 month to 20 years), only one case had local recurrence 9 years after initial incomplete excision (7), indicating a good prognosis and low recurrence rate, although long-term follow-up is still recommended (7, 11). The patient in this case study completed the treatment and remained in good condition without recurrence.

## Conclusions

SMF of the lower female genital tract is a relatively rare benign mesenchymal tumor most likely to occur in the vagina. The age range of SMF patients is wide; however, it mainly occurs in perimenopausal and postmenopausal women. Here, we report the diagnosis and treatment of superficial vaginal myofibroblastoma in a postmenopausal woman. For the first time, colposcopy was used for auxiliary diagnosis and evaluation before surgery. The lesion was covered with normal squamous epithelium with a wide pedicle and a mushroom-like appearance. The patient had a good prognosis and experienced no recurrence after surgical treatment.

## Data availability statement

The original contributions presented in the study are included in the article/supplementary material. Further inquiries can be directed to the corresponding author.

## Ethics statement

The studies involving human participants were reviewed and approved by Institutional Review Board of Shengjing Hospital of China Medical University. The patients/participants provided their written informed consent to participate in this study. Written informed consent was obtained from the individual(s) for the publication of any potentially identifiable images or data included in this article.

## Author contributions

YW was responsible for drafting of the manuscript. MS analyzed the literature. JW was responsible for the data collection and critical revision of the manuscript. All authors contributed to the article and approved the submitted version.

## Funding

This work was supported by the Shengjing Hospital of China Medical University 345 Talent Project (No. 30B).

## Acknowledgments

We would like to thank the patient for her permission to present this case report to sensitize practitioners. We also thank all the medical staff who participated in the diagnosis and treatment of this patient.

## Conflict of interest

The authors declare that the research was conducted in the absence of any commercial or financial relationships that could be construed as a potential conflict of interest.

## Publisher’s note

All claims expressed in this article are solely those of the authors and do not necessarily represent those of their affiliated organizations, or those of the publisher, the editors and the reviewers. Any product that may be evaluated in this article, or claim that may be made by its manufacturer, is not guaranteed or endorsed by the publisher.
